# Unveiling the activation dynamics of a fold-switch bacterial glycosyltransferase by ^19^F NMR

**DOI:** 10.1074/jbc.RA120.014162

**Published:** 2020-05-20

**Authors:** Jobst Liebau, Montse Tersa, Beatriz Trastoy, Joan Patrick, Ane Rodrigo-Unzueta, Francisco Corzana, Tobias Sparrman, Marcelo E. Guerin, Lena Mäler

**Affiliations:** 1 Department of Biochemistry and Biophysics, Stockholm University, Stockholm, Sweden; 2 Structural Biology Unit, Center for Cooperative Research in Biosciences (CIC bioGUNE), Basque Research and Technology Alliance (BRTA), Bizkaia Technology Park, Derio, Spain; 3 Departamento de Bioquímica and Instituto Biofisika, Consejo Superior de Investigaciones Científicas–Universidad del País Vasco/Euskal Herriko Unibertsitatea (CSIC, UPV/EHU), Bizkaia, Spain; 4 Departamento de Química, Centro de Investigación en Síntesis Química, Universidad de La Rioja, Logroño, Spain; 5 Department of Chemistry, Umeå University, Umeå, Sweden; 6 IKERBASQUE, Basque Foundation for Science, Bilbao, Spain

**Keywords:** protein structure, protein fold-switching, protein dynamics, conformational dynamics, protein function, enzyme catalysis, ^19^F NMR, relaxation dispersion, carbohydrate active enzymes, glycosyltransferases

## Abstract

Fold-switch pathways remodel the secondary structure topology of proteins in response to the cellular environment. It is a major challenge to understand the dynamics of these folding processes. Here, we conducted an in-depth analysis of the α-helix–to–β-strand and β-strand–to–α-helix transitions and domain motions displayed by the essential mannosyltransferase PimA from mycobacteria. Using ^19^F NMR, we identified four functionally relevant states of PimA that coexist in dynamic equilibria on millisecond-to-second timescales in solution. We discovered that fold-switching is a slow process, on the order of seconds, whereas domain motions occur simultaneously but are substantially faster, on the order of milliseconds. Strikingly, the addition of substrate accelerated the fold-switching dynamics of PimA. We propose a model in which the fold-switching dynamics constitute a mechanism for PimA activation.

Proteins do not just occupy a single state but are in dynamic exchange between a set of conformations, and some of these states may at times be populated at very low levels, so-called invisible or dark states (1–4). Motions occur on a broad range of timescales, and growing evidence suggests that dynamics are instrumental for the function of a multitude of proteins. In enzymatically mediated reactions, protein dynamics enable the recruitment and binding of substrates, stabilization of transition states, and adaptation to a dynamic membrane environment or binding partners (5, 6). Fold-switching, *i.e.* the interconversion of secondary structure elements, is a functionally essential dynamic event for a certain class of proteins. Such motions modify the topology of a protein, including α-helix–to–β-strand and/or β-strand–to–α-helix transitions (7–9). Alterations of such dynamic processes are essential for and causative of human disease, as in the case of the prion protein PrP and a variety of proteins associated with neurodegenerative diseases, including α-synuclein, amyloid β-peptide, and huntingtin (10, 11). Although it has been proposed that such major structural changes are widespread in proteins, structures of fold-switching proteins are underrepresented in the PDB (12), and studies of associated dynamics are scarce, remaining a major challenge (13–15).

Here, we report on the dynamics of the phosphatidyl-*myo*-inositol mannosyltransferase PimA, an essential enzyme in *Mycobacterium tuberculosis* (16, 17). PimA catalyzes the first step in the biosynthesis of phosphatidyl-*myo*-inositol mannosides (PIMs) by adding a mannose residue donated by the nucleotide sugar GDP-mannose (GDP-Man) to a phosphatidyl-*myo*-inositol lipid, which is anchored into the cytoplasmic phase of the plasma membrane (16, 18). PIMs are critical structural components of the mycobacterial cell envelope and precursors of two cell envelope lipoglycans, lipomannan and lipoarabinomannan (19, 20), both of which are virulence factors during tuberculosis (21), one of the deadliest infections worldwide (22). Crystal structures were previously obtained of PimA in the unliganded state (23) and in the presence of GDP or GDP-Man (Fig. 1*A*) (24). In the crystal structure of unliganded PimA, two distinct conformations of a region in the N-terminal domain, termed the reshuffling region (residues 118–163), are seen, suggesting that this region can undergo motions between a compact and an extended conformation, even in the absence of substrate (Fig. 1*A*) (23, 25). Moreover, the crystal structures of PimA in the presence of GDP or GDP-Man support the hypothesis that the reshuffling region displays fold-switching of secondary structure elements with both α-helix–to–β-strand and β-strand–to–α-helix transitions upon addition of substrate (Fig. 1*A*) (23). Fold-switching activates PimA, since PimA mutants locked in the unliganded conformation are inactive, while mutants locked in the substrate-bound conformation are active (23). Finally, the occurrence of an open-to-closed motion between the N- and C-terminal Rossmann fold domains has been predicted and experimentally demonstrated to occur in PimA and other GT-B glycosyltransferases (24–28).

**Figure 1.**
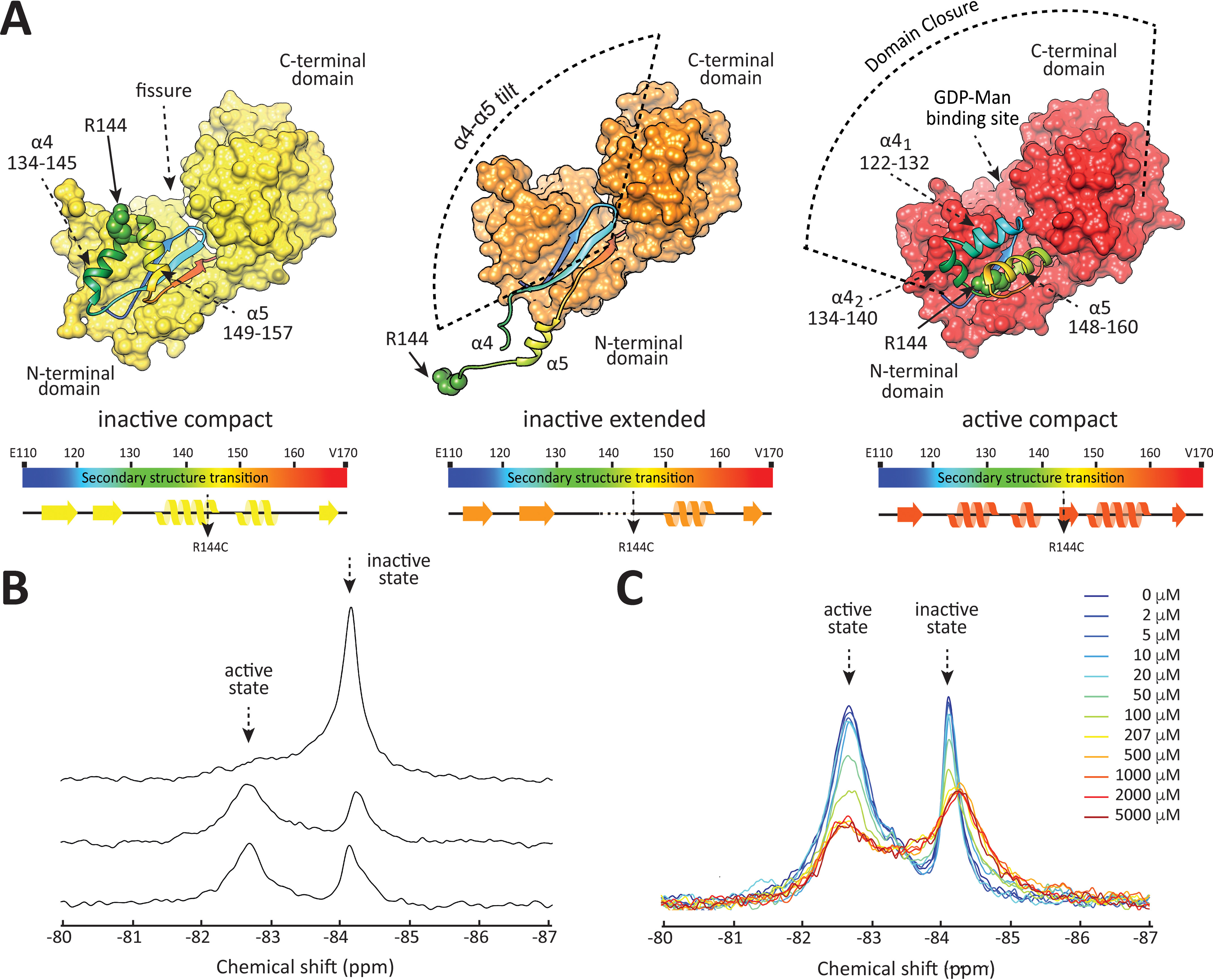
**Crystal structures of PimA.** (*A*) In its inactive state, PimA adopts two conformations (PDB entry 4NC9, apo state of PimA) with different tertiary structures in the reshuffling region (displayed in *rainbow color*) but identical topology. In the inactive, extended form (*orange*), two helices (α4 and α5) point away from the N-terminal domain and fold back along hinge loops onto the N-terminal domain in the inactive, compact conformation (*yellow*). Note that the loop connecting α4 and α5 is only partially resolved in the crystal structure of the inactive, extended state. Transition to the active state (PDB entry 2GEJ, PimA in the presence of GDP-Man) is accompanied by a fold-switch in the reshuffling region (*red*). In the crystal structures, R144 is shown/modeled as *spheres* in *green*. Selected secondary structure elements of PimA, including the reshuffling region (residues 118–163), as observed in the unliganded (inactive) compact and extended conformations and the liganded (active) PimA-GDP-Man complex, are shown. β5, β6, and β7 are shown in *blue*, *light blue*, and *orange*, respectively. (*B*) ^19^F spectra of PimA^T126C-V359C-R144C-TFA^ in 10% D_2_O (*top*), PimA^R144C-TFA^ in 100% D_2_O (*center*), and PimA^R144C-TFA^ in 10% D_2_O (*bottom*). (*C*) ^19^F spectra of PimA^R144C-TFA^ in the absence (*blue*) and at increasing concentrations of GDP-Man. Intensities are corrected for dilution effects.

Here, we demonstrate by using ^19^F NMR methods that motions of PimA occur on a second-to-millisecond timescale and include interconversions between several conformational states. Our observations provide a basis to suggest an activation mechanism for PimA in which the addition of substrate accelerates dynamics between active and inactive conformations.

## Results

### ^19^F labeling is a sensitive approach to study dynamics of PimA by NMR

^19^F nuclei are NMR active with a gyromagnetic ratio that is 0.94 times that of protons, have 100% natural abundance, and display high sensitivity to their magnetic environment (29–32). At the same time, fluorine is nearly absent from biological molecules, reducing the number of resonances observed in a spectrum to those that stem from artificially introduced ^19^F labels. Therefore, ^19^F nuclei are suitable probes that allow employing NMR experiments for dynamic studies even for large proteins for which more common labeling approaches are not successful (31). Based on analyses of the different crystallographic structures of PimA (Fig. 1*A*), we introduced a Cys mutation in position Arg144 (here termed PimA^R144C^) (Fig. 1*A*) and labeled it with 1,1,1-trifluoroacetone (TFA), giving rise to a ^19^F labeled mutant PimA^R144C-TFA^ (see Experimental procedures). The introduced ^19^F label of the mutant PimA^R144C-TFA^ is located in different chemical environments in the different crystallographic conformations of PimA, as highlighted in Fig. 1*A*. In the active state, the residue is buried inside the protein and, thus, not exposed to solvent, while it is solvent-exposed in the inactive states. ^19^F nuclei have been shown to be sensitive to such differences in the hydrophobicity of their immediate environment (32, 33). Thus, it can be expected that the ^19^F probe located at Cys144 can sense motions that we hypothesize to occur in the reshuffling region, which are 1) extended to compact domain motion and 2) fold-switching dynamics between the inactive and active states.

Given that the probe experiences sufficiently distinct chemical environments, domain motions that are typically on the order of milliseconds can be probed by NMR relaxation dispersion experiments (34). In addition, fold-switching dynamics are expected to occur on a second timescale and can be probed by saturation transfer experiments (35).

We first determined that the introduced label does not alter the three-dimensional structure of PimA. Supporting this notion, wild-type PimA, PimA^R144C^, and labeled PimA^R144C-TFA^ mutants displayed similar far-UV CD spectra (Fig. S1A) (25). In addition, the use of thermal unfolding experiments followed by CD has been extensively used to measure protein-ligand binding interaction (36–39). The midpoint temperature of unfolding for the unliganded protein is referred to as *T_m_*. It is expected that the addition of ligands to the protein sample will increase the *T_m_*. The higher the affinity of a ligand, the higher the apparent *T_m_* value, because it requires more energy to dissociate higher affinity compounds (39). We have previously described that the addition of GDP or GDP-Man to wild-type PimA increases the *T_m_* of the enzyme, with the β-phosphate of the nucleotide group playing a prominent role (25). As depicted in Fig. S1B, thermal unfolding followed by the far-UV CD signal at 222 nm indicated slight differences in protein stability between unliganded WT PimA, PimA^R144C^, and PimA^R144C-TFA^. Interestingly, after the addition of GDP, wild-type PimA, PimA^R144C^, and PimA^R144C-TFA^ displayed a clear increase in the *T_m_* values (Table S1), supporting the notion that the mutation and labeling do not significantly perturb the enzymatic structure and function (Fig. S1).

**Table 1:** **Parameters obtained from saturation transfer experiments for (*A*) the active state resonance and (*B*) for the inactive state resonance of PimA^R144C-TFA^**

Condition and resonance state	Chemical shift (ppm)	*k_IA_^a^* (s^−1^)	*p_I_*	*p_A_*	*k_s_* (s^−1^)	Δ*G_IA_* (kcal mol^−1^)
**Active state**						
Apo	−82.7	1.08 ± 0.05		0.47 ± 0.04	2.31 ± 0.09	−0.070 ± 0.090
GDP-Man	−82.6	4.2 ± 0.2		0.34 ± 0.04	13 ± 1	−0.390 ± 0.180
I**nactive state**						
Apo	−84.1	1.23 ± 0.04	0.53 ± 0.04		2.31 ± 0.09	0.070 ± 0.090
GDP-Man	−84.3	8.3 ± 0.9	0.66 ± 0.12		13 ± 1	0.390 ± 0.180

*^a^I*, the inactive state; *A*, the active state, *i.e. k_IA_* is the exchange rate from the inactive to the active state, and Δ*G_IA_* is the free energy difference between the inactive and active state and vice versa.

### Active and inactive states of PimA show distinct resonances

PimA contains two Cys residues buried inside the protein core that are not expected to be labeled during the labeling procedure (see Experimental procedures). To verify this, the labeling scheme was applied to WT PimA. Only resonances that are less than 5 Hz wide were observed in a ^19^F spectrum (Fig. S2), showing that they stem from small molecules. The resonances could be assigned to residual 3-bromo-1,1,1-trifluoroacetone (BTFA) label and 1,1,1-trifluoroacetone (TFA), the latter being the product of an incomplete labeling reaction. The resonance at −119.8 ppm stems from an impurity in the BTFA solution. Therefore, no labeling was observed for WT PimA. We conclude that PimA^R144C-TFA^ is only labeled at the introduced Cys residue.

**Figure 2.**
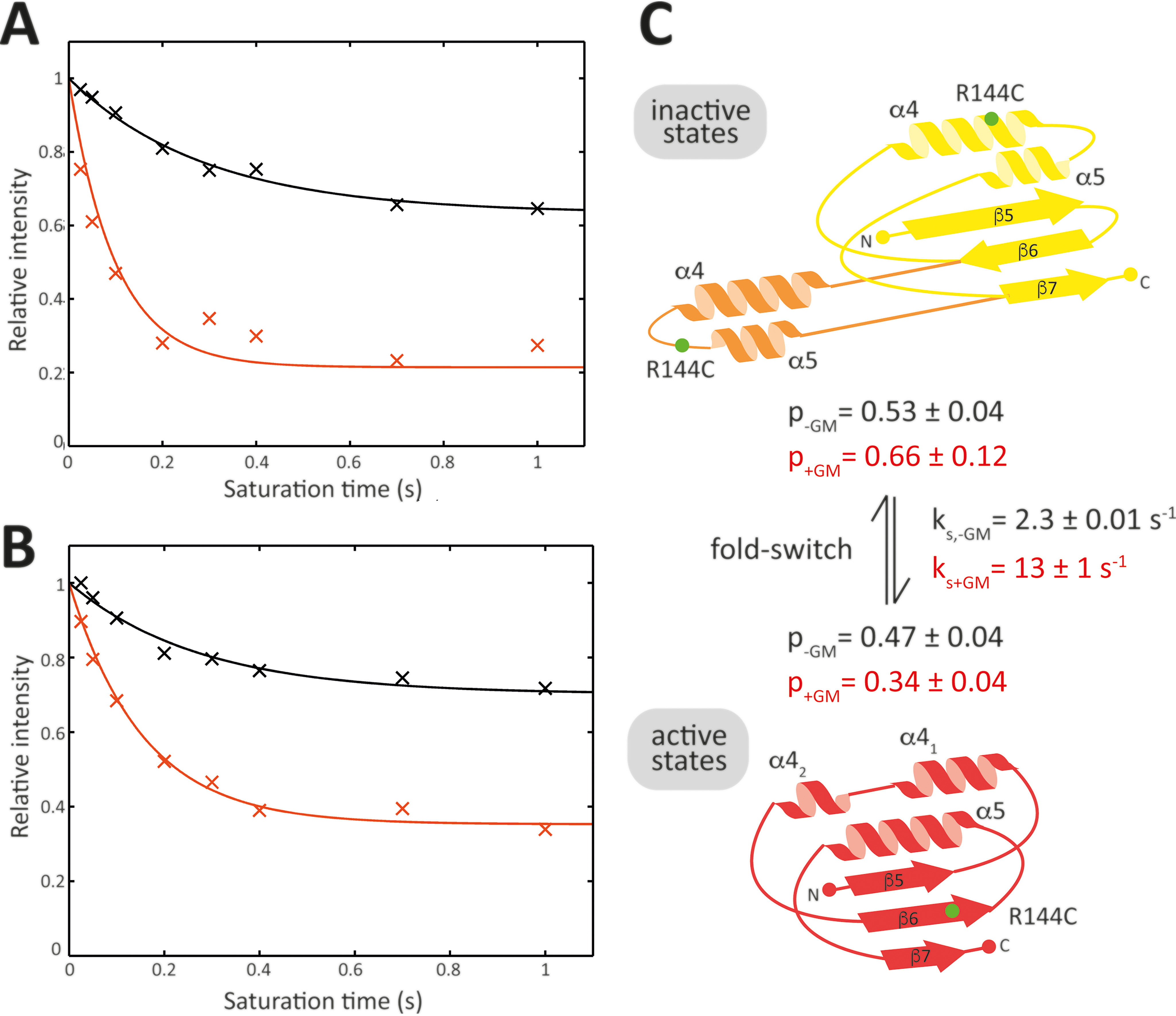
**Slow conformational exchange of PimA^R144C-TFA^.** (*A*) Intensity attenuation of the active state resonance in absence (*black*) and presence (*orange*) of saturating concentrations of GDP-Man upon saturation of the inactive state resonance. (*B*) Intensity attenuation of the inactive state resonance in the absence (*black*) and presence (*orange*) of saturating concentrations of GDP-Man upon the saturation of the active state resonance. Experiments were conducted with 100 μm PimA^R144C-TFA^ and 500 μm GDP-Man, where applicable. (*C*) Secondary structure representation of the reshuffling region. The position of the mutation (R144C) is indicated as a *green dot*. Parameters (p, populations; k_s_, slow exchange rates) are obtained from the fit to data in *panels A* and *B*.

Despite being singly labeled, two resonances are observed at −84.1 ppm and −82.7 ppm (Fig. 1*B*, *bottom*). As discussed below, these two peaks display saturation transfer, further corroborating that they stem from the same ^19^F label. To assign the resonances to conformations of PimA, we investigated the ^19^F spectrum of the triple mutant PimA^T126C-V359C-R144C-TFA^, previously shown to be locked in the inactive conformation. This variant cannot undergo reshuffling due to a disulfide bond formed between Cys126 and Cys359; these residues are in close spatial proximity in the inactive state but far apart in the active state (23). PimA^T126C-V359C-R144C-TFA^ displays only a single resonance at −84.1 ppm (Fig. 1*B*, top), which therefore can be assigned to the inactive state. Consequently, the resonance at −82.7 ppm is assigned to the active state. This assignment is corroborated by the fact that the resonance at −84.1 ppm shifts 0.1 ppm upfield in 100% D_2_O, whereas the resonance at −82.7 ppm does not shift (Fig. 1*B*, center). For fully solvent-exposed ^19^F labels, upfield solvent isotope shifts of 0.2–0.3 ppm have been observed when comparing shifts in a 90% H_2_O/10% D_2_O to 100% D_2_O solvent (33, 40), indicating that the resonance at −84.1 ppm stems from partially exposed ^19^F label. In the crystal structure, Arg144 is shielded from solvent in the active state, whereas this is not the case for the inactive conformations (Fig. 1*A*), supporting the assignment. The assignment is further supported by extensive molecular dynamics (MD) simulations performed on the different states of PimA labeled with TFA. Based on these simulations, we calculated the solvent-accessible surface area (SASA) of the TFA label in the different states. A higher SASA value indicates that the label is more solvent-exposed. As shown in Fig. S3, the SASA of the TFA label in the active state is 19.3 Å^2^, which differs markedly from the values obtained for the protein in the inactive forms (96.7 Å^2^ and 60.1 Å^2^ for the extended and compact structures, respectively). Thus, MD simulations revealed that the TFA label in the active state is less surface exposed than that in the inactive state, in agreement with the NMR experiments.

**Figure 3.**
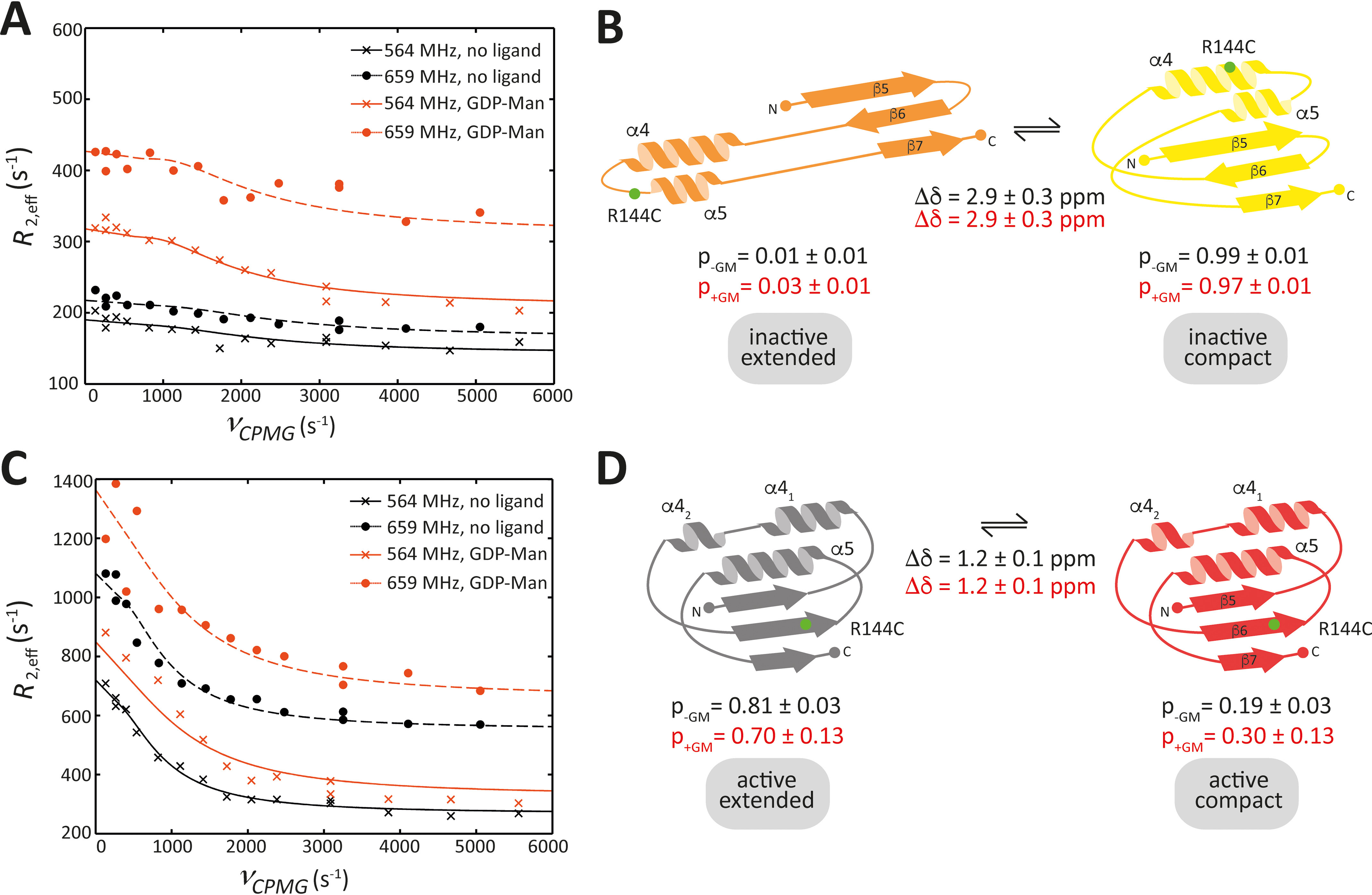
**Intermediate conformational exchange of the inactive and the active states of PimA^R144C-TFA^.** (*A*) Relaxation dispersion of the inactive state resonance in the absence (*black*) and presence (*orange*) of saturating concentrations of GDP-Man. (*B*) Extended-to-compact dynamics in the reshuffling region (extended conformation in *orange*; compact conformation in *yellow*). Parameters (p, populations; Δδ, chemical shift difference) obtained from the fit to data in *panel A* are indicated (*black*, no ligand; *orange*, with GDP-Man). (*C*) Relaxation dispersion of the active state resonance in the absence (*black*) and presence (*orange*) of saturating concentrations of GDP-Man. (*D*) Extended-to-compact dynamics that occur in the reshuffling region (extended conformation in *gray*; compact conformation in *red*). Only the compact, active conformation is structurally characterized. Parameters (p, populations; Δδ, chemical shift difference) obtained from the fit to the data in *panel C* are indicated (*black*, no ligand; *orange*, with GDP-Man). Experiments were conducted with 100 μm PimA^R144C-TFA^ and 500 μm GDP-Man where applicable. All observed exchange rates are on the order of 4000–8000 s^−1^ (see Table 1 for details).

### PimA undergoes slow conformational exchange between the inactive and active states

To test whether the two resonances in the ^19^F spectrum of PimA^R144C-TFA^ are in slow exchange, we conducted saturation transfer experiments at increasing saturation times. Exchange rates were obtained from a fit to the model given in Equations 4–7. Experimental data to obtain *R*_1_ longitudinal relaxation rates that are required for the analysis are shown in Fig. S4 and Table S2. Figure 2 shows resonance intensity attenuation due to slow conformational exchange for the inactive and active state resonances upon saturation of the active or inactive state resonance, respectively.

**Figure 4.**
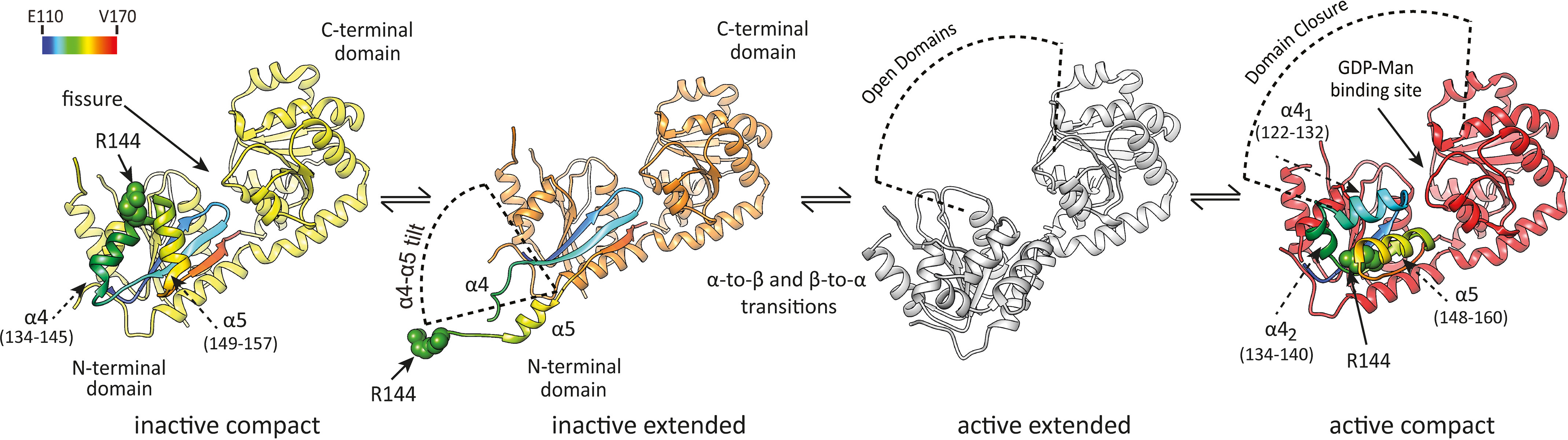
**Activation mechanism of PimA.** Structural changes of PimA upon activation. Initially, almost all inactive PimA molecules are in the compact conformation but are in millisecond exchange with the inactive, extended state. Following the fold-switch, PimA is in the structurally uncharacterized active, extended state and in millisecond exchange with the active, compact state, which is the conformation in which catalysis occurs. The location of R144 is shown/modeled for clarity.

**Table 2:** **Parameters obtained from CPMG relaxation dispersion experiments of PimA^R144C-TFA^ fitted to Equation 3**

Condition	Conformation	Chemical shift (ppm)	*k_i_* (10^3^ s^−1^)	*p*_extended_	*p*_compact_	|Δδ|*^a^* (ppm)
Apo	Active	−82.7	4.3 ± 1.3	0.81 ± 0.03	0.19 ± 0.03	1.2 ± 0.1
GDP-Man	Active	−82.6	7.3 ± 1.5	0.70 ± 0.13	0.30 ± 0.13	1.2 ± 0.1
Apo	Inactive	−84.1	8.0 ± 8.0	0.009 ± 0.002	0.991 ± 0.002	2.9 ± 0.3
GDP-Man	Inactive	−84.3	5.1 ± 2.7	0.026 ± 0.008	0.974 ± 0.008	2.9 ± 0.3

*^a^*|Δδ| is a global variable for each peak.

In the absence of GDP-Man, the exchange rate was determined to be 2.3 s^−1^. These dynamics correspond to fold-switching between the active and inactive states of the protein, as demonstrated by the assignments (Fig. 1*B*). The second timescale of the motion is in accordance with studies of the chemokine lymphotactin, for which fold-switching dynamics were shown to be on the order of seconds (14). In the absence of GDP-Man, the active and inactive states of PimA are nearly equally populated (Fig. 2*C* and Table 1).

To test how GDP-Man affects these dynamics, we first conducted a titration experiment to corroborate that the sugar donor saturates the binding site. As shown in Fig. 1*C*, titration of GDP-Man to 100 μm PimA^R144C-TFA^ leads to a gradual decrease in intensities of both resonances up to a nucleotide sugar concentration of 200 μm. At this concentration, the inactive state resonance shifts 0.2 ppm upfield, and further addition of GDP-Man does not alter the spectrum anymore. At the field strength employed in Fig. 1*C* (16.4 T), the active state resonance is too broad to clearly observe a resonance shift. Since the line broadening due to the field-dependent chemical shift anisotropy of ^19^F is reduced at lower field strength, we compared the spectra of PimA^R144C-TFA^ in the presence and absence of GDP-Man at 14.1 T. At this lower field, a downfield shift of the active state resonance of 0.1 ppm was observable at 500 μm GDP-Man and 100 μm PimA^R144C-TFA^ (Fig. S5). Moreover, both peaks broaden upon addition of GDP-Man, indicating that the presence of GDP-Man alters dynamics and/or populations of PimA. This process may be related to GDP-Man binding itself or to the dynamic processes observed in the reshuffling region of PimA, as discussed below. GDP-Man and GDP were previously seen to bind PimA in enthalpy-driven reactions with dissociation constants (*K_D_*) of 0.23 μm (Δ*H* = –15.6 kcal·mol^−1^) and 0.03 μm (ΔH= –14.0 kcal·mol^−1^), respectively (41). Thus, the submicromolar *K_D_* indicates that under the experimental conditions employed here, 500 μm GDP-Man and 100 μm PimA^R144C-TFA^ binding of GDP-Man to PimA is fully saturated. Thus, we can exclude any contribution of GDP-Man binding to the observed dynamics discussed subsequently.

Under the condition that GDP-Man is present at saturating concentrations, the fold-switching dynamics of PimA^R144C-TFA^ are strongly enhanced, and the exchange rate was observed to increase to 13 s^−1^ (Fig. 2 and Table 1). A concurrent shift in the population equilibrium toward the inactive states was observed (by around 10%), as evidenced by analyzing peak integrals as well as the saturation transfer experiments (Fig. 2, Fig. S5, and Table S3).

### The inactive state of PimA undergoes millisecond motions between a compact and an extended state

To capture possible dynamics on the millisecond timescale, we conducted Carr-Purcell-Meiboom-Gill (CPMG) relaxation dispersion experiments on PimA^R144C-TFA^ at two magnetic field strengths in the absence and presence of saturating amounts of GDP-Man. Assuming a two-state exchange, we fitted the data to Equation 3 (see Experimental procedures) to obtain an estimate of the size of the populations, the exchange rate between them, and the chemical shift difference due to the conformational exchange. Data that were acquired at one magnetic field strength only were fitted to Equation 2 to obtain an apparent exchange rate.

The apparent exchange rate of the resonance representing the inactive state of PimA^R144C-TFA^ and the triple mutant PimA^T126C-V359C-R144C-TFA^ locked into the inactive state are identical (Fig. S6). Although the triple mutant cannot undergo reshuffling to the active state, the faster domain dynamics of the inactive state itself are not affected. This is expected from inspecting the structure of PimA. While the disulfide bond between Cys126 and Cys359 prevents secondary structure reshuffling, it does not impede the extended-to-compact motion of the inactive states (Fig. 1*A*). Thus, the relaxation dispersion displayed by the inactive state resonance monitors the extended-to-compact dynamics of the reshuffling region.

As shown in Fig. 3*A*, the resonance in the ^19^F spectrum of PimA^R144C-TFA^ monitoring the inactive state displays relaxation dispersion between two conformations with different populations, and the profile is affected by the addition of saturating amounts of GDP-Man, reflecting changes in dynamics and population equilibria. In the absence of GDP-Man, the exchange rate is 8000 s^−1^, which decreases to 5100 s^−1^ in the presence of GDP-Man (Table 2). Similar exchange rates have previously been observed for motions of a helix of the β2-adrenergic receptor (32). In the absence of GDP-Man, one of the states is highly populated and the other one is not (1% *versus* 99%). Upon addition of GDP-Man, the less populated of the inactive states becomes slightly more populated (3% *versus* 97%), which is accompanied by an upfield resonance shift of the inactive state resonance (Fig. 1*C*), indicating that the observed change in populations and dynamics results in a more polar environment, on average, for the ^19^F label. While the population change upon addition of GDP-Man (1.7%) as determined from the relaxation dispersion data is significant, given the fitted errors in the analysis of the relaxation data (Table 2), it might not be possible to deconvolute model parameters entirely, given that experiments were acquired at two magnetic field strengths only; however, changes in dynamics and changes in populations toward the less populated state are also manifest in the observed upshift of the inactive state resonance in Fig. 1*C*, *i.e.* to the state in which the ^19^F label is located in a more polar environment. Resonances of ^19^F nuclei in a more polar environment are located upfield of ^19^F nuclei buried inside a protein (32, 33). This is in agreement with our observation that the inactive state resonance, for which the ^19^F label is more exposed to the solvent than for the active state resonance (Fig. 1*B*), is upfield of the active state resonance. Based on the crystal structures (Fig. 1*A*) and our MD simulations (Fig. S3), the ^19^F label is most solvent-exposed in the inactive, extended conformation. Thus, we identify the sparsely populated state with the inactive, extended conformation. This conclusion is further corroborated by structural features of PimA.

The conformational transition from the compact to extended inactive state of PimA, in the absence of GDP-Man, was reproduced by steered-MD simulations, as shown in Fig. S7. This type of calculation forces the system to evolve away from its initial equilibrium condition (compact, inactive) to a final state (extended, inactive), thereby accelerating transitions between different energy minima (42–44). This method is generally applied in studying many biophysical processes, including the (un)folding mechanism of proteins (45, 46). As revealed by the steered-MD simulations, the inactive, extended state's large hydrophobic patches are exposed, making this state potentially aggregation-prone, whereas the hydrophobic patches are shielded from solvent in the inactive, compact state (23). The fact that the addition of GDP-Man leads to an increase in population of the inactive, extended state suggests that the slower reshuffling event occurs in that state.

### The active state of PimA undergoes millisecond motions that may reflect open-to-closed dynamics

As shown in Fig. 3*C*, the resonance in the ^19^F spectrum of PimA^R144C-TFA^ monitoring the active states also displays relaxation dispersion, and the profile is again affected by the addition of saturating amounts of GDP-Man, reflecting changes in dynamics and population equilibria. In contrast to the inactive state resonance, the active state resonance is downshifted upon addition of GDP-Man (Fig. S5). This indicates that a population shift toward a more hydrophobic environment for the ^19^F label occurs, which might be identified with a population shift from a structurally uncharacterized active, extended state to the active, compact state of PimA. Based on this interpretation, most of PimA is found in the active, extended state (81% extended *versus* 19% compact). This holds true even after addition of GDP-Man (70% extended *versus* 30% compact) (Fig. 3*D*). The active, extended state might be represented by the open version of the already structurally characterized active, closed state, suggesting that these dynamics correspond to the open-to-closed motion of PimA (Fig. 1*A*). The exchange rate between the extended and compact forms of the active states in this case increases from 4300 s^−1^ to 8000 s^−1^ upon addition of saturating concentrations of GDP-Man.

Interestingly, accelerated MD simulations performed on PimA labeled with TFA and starting from the compact, active state indicated that the protein undergoes a conformational change to reach a state in which β7 dissociates from β6, which indicates the initiation of the transition to α-helix (Fig. S8). As steered-MD simulations, this computational approach is useful for improving conformational space sampling. The method does not require any predefined coordinates and reduces the energy barrier between the different low-energy states (47).

### The active and inactive states are structurally distinct

The relaxation dispersion data indicate that the inactive, compact and inactive, extended states are structurally distinct, as their resonances are separated by 1.2 ppm (Table 2). As discussed above, the resonance of the inactive, extended state is expected to be upfield of the resonance of the inactive, compact state, since the ^19^F label is more solvent-exposed in the latter (Fig. 1*C*). Similarly, the relaxation dispersion data indicate that the active, compact and active, extended state are structurally distinct, as their resonances are separated by 2.9 ppm (Table 2). The downfield shift of the resonance of the active, compact state compared with the active, extended state (Fig. S5) indicates that in the active, compact state the ^19^F label is located in a more hydrophobic environment. Importantly, from the relaxation dispersion data (Table 2) and the 1D spectra (Fig. 1*C*, Fig. S5), it can be concluded that all conformational states are structurally distinct, indicating that fold-switching is accompanied by a dramatic structural rearrangement near the ^19^F label. This is consistent with the structural data that show that residue Arg144 is reshuffled from an α-helix (potentially via a solvent-exposed random coil structure) to a β-sheet environment.

## Discussion

The ability of an enzyme to adopt many different conformations during the catalytic cycle is due to its great flexibility, playing a critical role in the recruitment of substrates, release of products, stabilization of transition states, and adaptation to the cell environment. Employing ^19^F NMR, we identified four functionally relevant states of PimA that coexist in dynamic equilibria in solution undergoing conformational exchange on timescales from milliseconds to seconds. Specifically, fold-switching, a hitherto poorly characterized process, occurs in the reshuffling region of the N-terminal domain of PimA on a second timescale. Domain motions, on the other hand, occur on a millisecond timescale.

The observations presented here allow us to propose that the activation of PimA occurs along a sequence of structural rearrangements displayed in Fig. 4. In Fig. S9, a free-energy diagram of the activation process is depicted that is derived from thermodynamic data obtained from the saturation transfer and relaxation dispersion experiments (Tables 1 and 2). Initially, PimA is inactive and in a compact state that shields hydrophobic patches in the reshuffling region. Almost all inactive PimA molecules are in that conformation but are in millisecond exchange with the inactive, extended state. This state exposes hydrophobic patches and, thus, is sparsely populated. However, it offers sufficient degrees of freedom for a rearrangement of secondary structure elements on a second timescale. A drastic change in chemical shift indicates that the inactive, extended state is structurally distinct from the uncharacterized active, extended state, which might correspond to the open state of the structurally characterized closed, GDP-Man-bound conformation of PimA. Following the fold-switch, which we speculate to occur from the inactive, extended state, PimA is in the active, extended state and in millisecond exchange with the active, compact state, which is the conformation in which catalysis occurs. We propose that fold-switching is an activation mechanism of PimA, and since it is a slow process, it may represent the rate-limiting step of the glycosylation reaction catalyzed by PimA. The substrate, GDP-Man, acts as an activator and, thus, functional regulator of PimA, as the binding of GDP-Man enhances the fold-switching dynamics. Intriguingly, a significant population of the inactive state is always present. This may point to a more significant role of the inactive states in the catalytic cycle of PimA than previously anticipated, for instance, in membrane binding of PimA or to facilitate efficient recruitment of phosphatidyl-*myo*-inositol lipids, which are acceptor substrates of PimA.

### Conclusions

This study demonstrates that the ^19^F-based NMR approach presented here allows for insights into structures and dynamics for proteins that cannot be studied with more conventional NMR methods. The methods employed here, a combination of saturation transfer methods and relaxation dispersion experiments, allow investigations of dynamics occurring simultaneously and on a wide range of timescales in solution. Here, this approach revealed a remarkable situation in which PimA exists in at least four different conformations that are all present in solution. The dynamics between and the relative populations of these conformations are affected by the addition of substrate, which demonstrates that the equilibria are finely tuned to optimize the catalytic cycle.

## Materials and Methods

### Materials

1,1,1-Trifluoroacetone (TFA), 3-bromo-1,1,1-trifluoroacetone (BTFA), guanosine 5′-diphospho-d-mannose (GDP-Man), and tris(2-carboxyethyl)phosphine (TCEP) were acquired from Merck (Darmstadt, Germany).

### Cloning and purification of WT PimA and PimA mutants

PimA^R144C^ and PimA^T126C-V359C-R144C^ mutants of PimA from *Mycobacterium smegmatis* were obtained from GenScript using pET29a-*pimA* as the DNA template (25). Recombinant WT PimA and the PimA^R144C^ mutant were expressed in *Escherichia coli* BL21(DE3) pLysS and purified as described previously (25). PimA^T126C-V359C-R144C^ mutant was expressed in *E. coli* Rosetta Gami 2(DE3) pLysS and purified as described previously (23).

### Labeling of PimA

An established protocol was adapted to label PimA with TFA (48). 1 mm TCEP was added to 100–200 μm PimA and incubated for 1 h on ice, except for PimA^T126C-V359C-R144C-TFA^, to which no TCEP was added. Next, BTFA was added in 10-fold molar excess (1–2 mm), and the solution was incubated overnight at 4 °C under gentle agitation. Finally, the solution was passed through a desalting column equilibrated with 50 mm Tris-HCl, pH 7.5, and 150 mm NaCl. The sample conditions for NMR experiments were 100 μm PimA, 50 mm Tris-HCl, pH 7.5, 150 mm NaCl, and 10% D_2_O. 500 μm GDP-Man was added where appropriate and if not stated otherwise.

To exchange to 100% D_2_O solvent, 100 μm PimA was concentrated roughly five times and diluted back to a concentration of 100 μm using 50 mm Tris-HCl, 150 mm NaCl in 100% D_2_O. This procedure was repeated 5 times.

### NMR spectroscopy

Experiments were performed at 25 °C on a Bruker Avance 600 MHz III HD spectrometer (^19^F Larmor frequency of 564 MHz). Relaxation dispersion and titration data for PimA^R144C-TFA^ were additionally acquired on a Bruker Avance 700 MHz spectrometer (^19^F Larmor frequency of 659 MHz). Both spectrometers were equipped with a ^19^F cryoprobe. Typical π/2 pulse lengths were 15–19 µs, and the acquisition time was 0.03 s. 10% D_2_O was added to the samples for frequency locking. For 1D spectra, 400 transients were acquired with a relaxation delay of 1 s. Data were processed in Topspin (Bruker, Billerica, MA) and analyzed using in-house Matlab routines (MathsWorks, Natick, MA) and Igor Pro (WaveMetrics, Lake Oswego, OR), employing an in-house script for nonlinear fitting of relaxation dispersion data (49). Overlapping resonances were deconvoluted, employing iNMR software (MestreLab Research, Spain) by fitting spectra to double-Lorentzian functions.

### Far-UV CD analysis

Spectra were acquired in a J-810 CD spectropolarimeter (Jasco Corp., Tokyo, Japan) by using Hellma 105.200-QS quartz cuvettes with a 1 cm optical path. Spectra were recorded in a continuous mode with 1 nm bandwidth, 1 s response, and a scan speed of 100 nm/min^−1^. Samples were 2.5 μm PimA, PimA^R144C^, or PimA^R144C-TFA^ in 10 mm Tris-HCl, pH 7.5. GDP was added at a 1:15 ratio, and 25 scans were accumulated to obtain the final spectra, which were further corrected for the baseline signal. Spectra were recorded in the 200–250 nm range at 20 °C.

### Temperature scans

Spectra were acquired in a J-810 CD spectropolarimeter (Jasco Corp., Tokyo, Japan) by using Hellma 110-QS quartz cuvettes with a 1 mm optical path by using a Peltier thermal device, allowing temperature control during the experiments. Spectra were recorded in a continuous mode with 1 nm bandwidth, 1 s response, and a scan speed of 100 nm/min^−1^. Samples were 10 μm PimA, PimA^R144C^, or PimA^R144C-TFA^ in 10 mm Tris-HCl, pH 7.5. Substrates were added at a 1:15 ratio. In this case, thermal dependencies of the ellipticity were monitored in the range from 20 °C to 90 °C at 222 nm. Temperature was increased stepwise by 1 °C/min.

### Relaxation dispersion experiments

1D ^19^F CPMG relaxation dispersion experiments (34, 50) were performed analogously to previous studies (32). 2400 transients were acquired during a constant time delay, *T*_CPMG_, of 3.84 ms for 14 refocusing frequencies, ν_CPMG_, ranging between ∼130 s^−1^ and ∼6000 s^−1^. Effective transverse relaxation rates, *R*_2,eff_, were computed from peak intensities *I*(ν_CPMG_) relative to the peak intensity, *I*_0_, in the absence of a constant time delay as follows

(Eq. 1) $$R_{2,eff}={-T}_{CPMG}^{-1}ln\left(\frac{I\left(v_{CPMG}\right)}{I_{0}}\right)$$

For data measured at one magnetic field strength, relaxation dispersion data were fitted to Luz-Meiboom's two-state model to obtain an apparent exchange rate, *k*_app_, of the dynamics (50):

(Eq. 2) $$R_{2,eff}\left(\nu_{CPMG}\right)=\frac{c}{k_{app}}\left(1-\frac{4\nu_{CPMG}}{k_{app}}tanh\left(\frac{k_{app}}{4\nu_{CPMG}}\right)\right)\;+\;R_{2\text{,}0}$$

where $\nu_{CPMG}=0.5\tau_{CPMG}^{-1}$, τ_CPMG_ is the separation between two 180° pulses and *k*_app_, *R*_2,0_, and $c=p_{A}p_{B}{\Delta \omega }^{2}$are parameters of the fit. The populations, *p_A_* and *p_B_*, of the two states and the chemical shift difference between the resonances, Δω, cannot be deconvoluted with this model.

Relaxation dispersion data measured at two magnetic field strengths were fitted to Carver-Richards model assuming two states to obtain the exchange rate of the process, *k_i_* (*i* indicates intermediate exchange), populations, *p_A_* and *p_B_*, and the chemical shift difference, Δω, between the states (34):

$$p_{A}=1-p_{B}$$

$$\xi =2\Delta \omega \left(p_{B}-p_{A}\right)k_{i}$$

$$\psi ={\left(p_{B}-p_{A}\right)}^{2}{k_{i}}^{2}-{\Delta \omega }^{2}\;+\;4p_{B}p_{A}{k_{i}}^{2}$$

$$\eta_{\pm }=\;\frac{1}{\sqrt{8}\nu_{CPMG}}\sqrt{\pm \psi \;+\;\sqrt{\psi^{2}\;+\;\xi^{2}}}$$

$$D_{\pm }=\frac{1}{2}\left(\pm 1\;+\;\frac{\psi \;+\;2{\Delta \omega }^{2}}{\sqrt{\psi^{2}\;+\;\xi^{2}}}\right)$$

(Eq. 3) $$R_{2,eff}=R_{2,0}+\frac{1}{2}\left(k_{i}-2\nu_{CPMG}\;arcosh\left(D_{+}\;cosh\left(\eta_{+}\right)-\;D_{-}cos\left(\eta_{-}\right)\right)\right)$$

To reduce the number of parameters in the fit, it was assumed that the addition of GDP-Man did not alter the conformations of PimA present in solution, *i.e.* the chemical shift $\Delta \delta =\Delta \omega /\left(2\pi f\right)$, where *f* is the spectrometer frequency, was treated as a global constant. Moreover, the magnetic field strength does not affect protein kinetics, *i.e. k_i_*, *p_A_*, and *p_B_* were identical for data obtained at different magnetic field strengths but could vary between samples that contained or did not contain GDP-Man.

### *T*_1_ *experiments*

Inversion recovery experiments with at least 10 delays, τ, ranging between 25 ms and 800 ms were performed to obtain the longitudinal relaxation time, *T*_1_. 1200 transients were acquired, and the relaxation delay was 2 s. The relaxation rate, *R*_1_ = *T*_1_^−1^, was obtained from a monoexponential fit of the intensities in dependence on delay τ.

### Saturation transfer experiments

Saturation transfer experiments were conducted to quantify slow exchange dynamics of PimA (32, 51). Resonances were saturated with continuous wave irradiation (B_1_ field of 28 Hz). The resonance associated with the active state was irradiated at −82.3 ppm, and the intensity decay of the inactive state resonance was monitored at −84.1 ppm in the absence of substrate and at −84.3 ppm in the presence of GDP-Man. Conversely, the resonance associated with the inactive state was irradiated at −84.3 ppm, and the intensity decay of the active state resonance was monitored at −82.7 ppm in the absence of substrate and at −82.6 ppm in the presence of GDP-Man. Spectra were acquired for 8 saturation times, varying between 25 ms and 1000 ms. 1200 transients were recorded, and the relaxation delay was 1.5 s. Exchange rates were obtained by fitting residual intensities, $I\left(\tau \right)$, at increasing irradiation times τ to a two-state model (35):

(Eq. 4) $$I_{m}\left(\tau \right)=\frac{I_{m}\left(0\right)}{k_{mn}+R_{1,m}}\left(k_{mn}exp\left(-\tau \left(k_{mn}+R_{1,m}\right)\right)+R_{1,m}\right)$$

Where *m* is inactive and *n* is active, representing the exchange rate, *k_mn_*, from the inactive to the active state and vice versa.

The exchange rate for slow dynamics, *k_s_* (*s* indicates slow exchange), is the sum of *k_mn_* and *k_nm_*:

(Eq. 5) $$k_{s}=k_{mn}+k_{nm}$$

Populations, *p*, of the states and the free-energy difference, Δ*G*, are given by

(Eq. 6) $$p_{n}=\frac{k_{mn}}{k_{s}}$$

(Eq. 7) $${\Delta G}_{nm}=-RT\;ln\frac{p_{n}}{p_{m}}$$

where *T* is the absolute temperature and *R* is the universal gas constant.

### Conventional MD simulations

The MD simulations were performed with the AMBER 18 package (52). Three different simulations, starting from the active-compact, inactive-compact, and inactive-extended conformations, were performed. Parameters for the TFA label were generated with the antechamber module of AMBER implemented with the general Amber force field (GAFF) (53), with partial charges set to fit the electrostatic potential generated with HF/6-31G(d) by RESP (54). The charges were calculated according to the Merz-Singh-Kollman scheme using Gaussian 09 (55). Each protein was immersed in a water box with a 10 Å buffer of TIP3P (56) water molecules. The system was neutralized by adding explicit counter ions (Na^+^). A two-stage geometry optimization approach was performed. The first stage minimizes only the positions of solvent molecules and ions, and the second stage is an unrestrained minimization of all the atoms in the simulation cell. The systems then were gently heated by incrementally increasing the temperature from 0 to 300 K under a constant pressure of 1 atm and periodic boundary conditions. Harmonic restraints of 30 kcal·mol^−1^ were applied to the solute, and the Andersen temperature coupling scheme (57) was used to control and equalize the temperature. The time step was kept at 1 fs during the heating stages, allowing potential inhomogeneities to self-adjust. Water molecules are treated with the SHAKE algorithm such that the angle between the hydrogen atoms is kept fixed. Long-range electrostatic effects are modelled using the particle-mesh-Ewald method (58). An 8 Å cutoff was applied to Lennard-Jones and electrostatic interactions. Each system was equilibrated for 2 ns with a 2 fs time step at a constant volume and temperature of 300 K. Production trajectories were then run for an additional 200 ns under the same simulation conditions. SASA for fluorine atoms of the TFA label was calculated using the keyword “SURF” in the *cpptraj* module (59) of AMBER.

### Accelerated MD simulations

With the accelerated MD implemented in AMBER 18, 500 ns accelerated MD simulations were performed on PimA labeled with TFA and starting from the active-compact conformation. These simulations were restarted from the final structure of the 200 ns conventional MD simulations with random atomic velocity initializations at 300 K. Boost potential with an extra boost to the torsions was applied (*iamd* = 3).

### Steered-MD simulations

The protocol employed above for conventional MD simulations was also used in steered-MD calculations (60). The compact-inactive conformation was used as the starting structure. For the final production trajectory, the distance between C-alpha of Ala148 and Ala366 was varied from 13.5 (close, inactive state, 0 ns) to 51.3 Å (extended, inactive state, 200 ns), using an *rk2* of 8 kcal·mol^−1^·Å^−1^.

## Data availability

All data described in the manuscript are contained within the manuscript or the supporting information.

## Supplementary Material

Supporting Information
