# Supplementary material for: Unveiling the activation dynamics of a fold-switch bacterial glycosyltransferase by 19F NMR
Source: J Biol Chem. 2020 May 20;295(29):9868–78. doi: 10.1074/jbc.RA120.014162 (PMC7380196; doi:10.1074/jbc.RA120.014162)
Supplement: Supporting Information [file supp_295_29_9868__index.html]

Unveiling the activation dynamics of a fold-switch bacterial glycosyltransferase by 19F NMR — Activation dynamics of a fold-switch glycosyltransferase — Unveiling the activation dynamics of a fold-switch bacterial glycosyltransferase by 19F NMR — Activation dynamics of a fold-switch glycosyltransferase — Supporting Information 

# Unveiling the activation dynamics of a fold-switch bacterial glycosyltransferase by 19F NMR

## Supporting Information

- Supporting Information (to be published online) - Supporting Information.
