# Supplementary material for: Unveiling the activation dynamics of a fold-switch bacterial glycosyltransferase by 19F NMR
Source: J Biol Chem. 2020 May 20;295(29):9868–78. doi: 10.1074/jbc.RA120.014162 (PMC7380196; doi:10.1074/jbc.RA120.014162)
Supplement: Supporting Information [file supp_RA120.014162_160566_1_supp_532989_qfkfg7.pdf]

# SUPPLEMENTAL INFORMATION

## UNVEILING THE ACTIVATION DYNAMICS OF A FOLD-SWITCH GLYCOSYLTRANSFERASE BY $^{19}$F NMR

**Jobst Liebau[1,†], Montse Tersa[2,†], Beatriz Trastoy[2], Joan Patrick[1], Ane Rodrigo-Unzueta[3], Francisco Corzana[4], Tobias Sparrman[5], Marcelo E. Guerin[2,3,6,‡], and Lena Mäler[1,5,‡]**

[1] Department of Biochemistry and Biophysics, Stockholm University, 106 91 Stockholm, Sweden
[2] Structural Biology Unit, CIC bioGUNE, Bizkaia Technology Park, 48160 Derio, Spain
[3] Instituto Biofisika, Consejo Superior de Investigaciones Científicas – Universidad del País Vasco/Euskal Herriko Unibertsitatea (CSIC,UPV/EHU), Barrio Sarriena s/n, Leioa, 48940 Bizkaia, Spain; Departamento de Bioquímica, Universidad del País Vasco, Leioa, 48940 Bizkaia, Spain
[4] Departamento de Química, Centro de Investigación en Síntesis Química, Universidad de La Rioja, 26006 Logroño, Spain
[5] Department of Chemistry, Umeå University, 901 87 Umeå, Sweden
[6] IKERBASQUE, Basque Foundation for Science, 48013 Bilbao, Spain

Running title: *Activation dynamics of a fold-switch glycosyltransferase*

*Keywords: protein structure, protein fold-switching, protein dynamics, conformational dynamics, protein function, enzyme catalysis, $^{19}$F NMR, relaxation dispersion, carbohydrate active enzymes, glycosyltransferases*

† These authors contributed equally

*To whom correspondence should be addressed: Marcelo E. Guerin, Structural Biology Unit, CIC bioGUNE, Bizkaia Technology Park, 48160, Derio, Spain, mrcguerin@cicbiogune.es; Lena Mäler, Department of Biochemistry and Biophysics, Stockholm University, 106 91 Stockholm, Sweden, lena.maler@dbb.su.se.

# SUPPLEMENTARY FIGURES

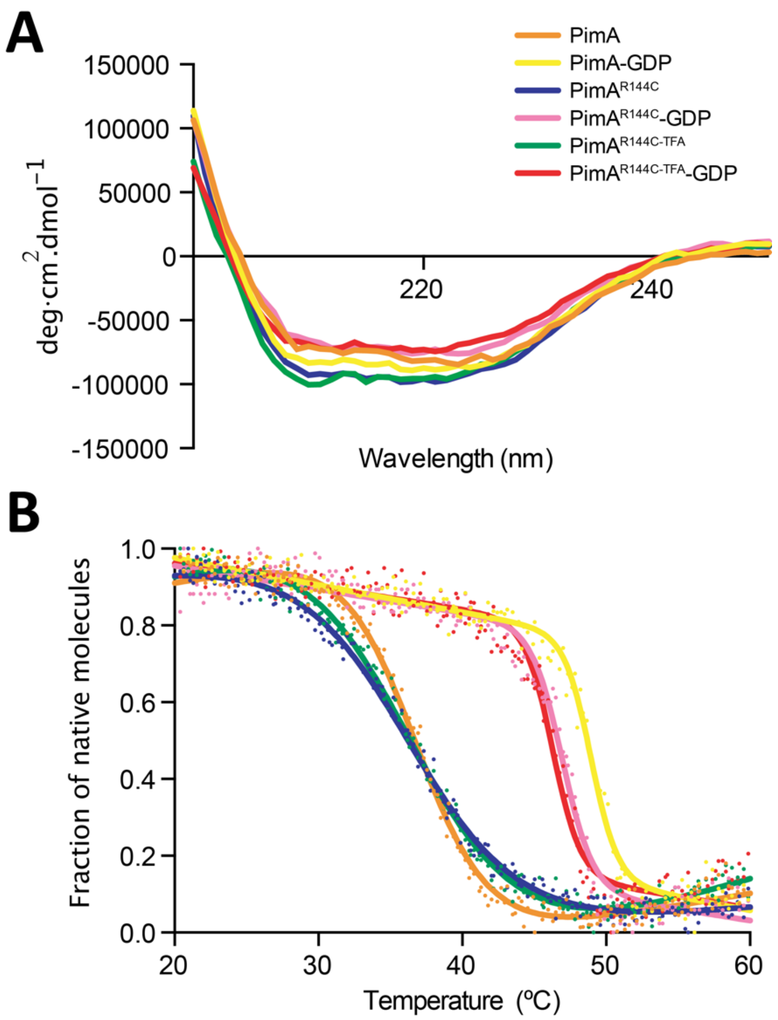


**Figure S1. CD spectroscopy of wild-type PimA, PimA$^{R144C}$ and PimA$^{R144C\text{-}TFA}$.** *A*. Far-UV CD spectra for wild-type PimA, PimA$^{R144C}$ and PimA$^{R144C\text{-}TFA}$ in the absence or presence of GDP. *B*. Thermal unfolding transitions of wild-type PimA, PimA$^{R144C}$ and PimA$^{R144C\text{-}TFA}$ monitored by the change in CD signal at 222 nm. The transition was tentatively fitted according to a two-state equilibrium model (solid lines) and normalized.

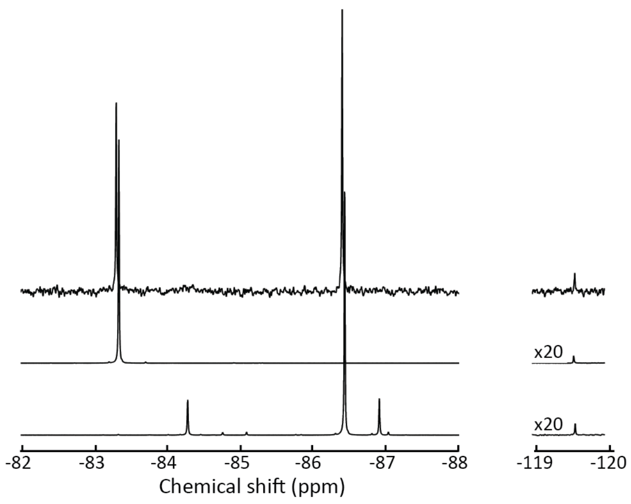


**Figure S2.** $^{19}$F spectra of *wild-type* PimA subjected to the labeling protocol (top), 10 mM BTFA in buffer (center), and 10 mM TFA in buffer (bottom). The intensity of the BTFA and TFA resonance at -119.8 ppm is 20-fold enhanced.

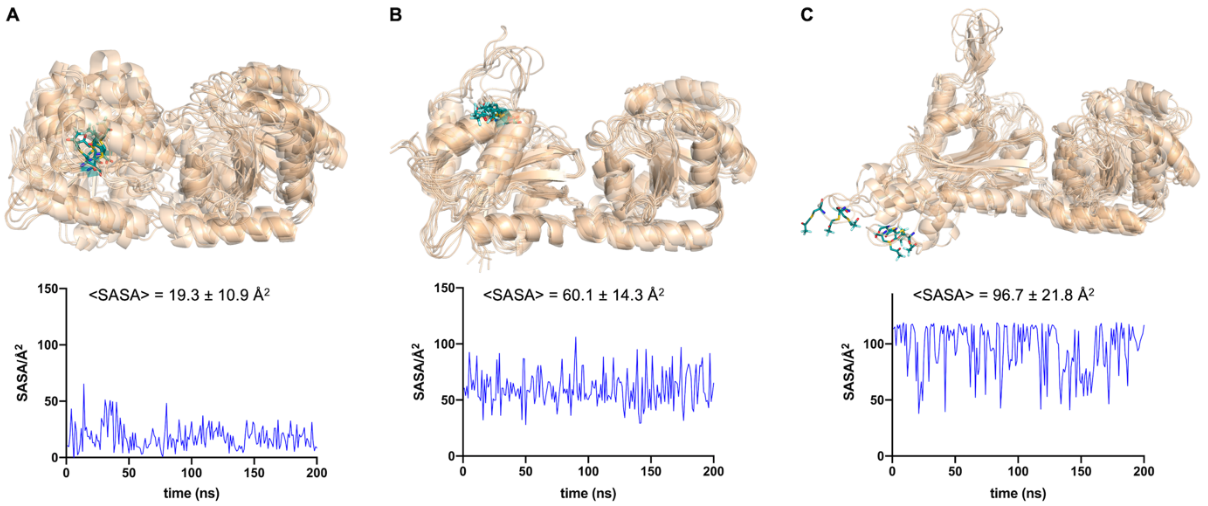


**Figure S3. MD simulations on PimA labeled with TFA.** Structural ensembles derived from 200 ns MD simulations for active-compact (*A*), inactive-compact (*B*) and inactive-extended (*C*) conformations, together with the evolution of solvent-accessible surface area (SASA) of the TFA label. The protein is shown as ribbons and the label as sticks. Hydrogen atoms have been removed for clarity. As can be seen, the label in the active state is less surface-exposed than in the inactive state, which is in agreement with the NMR experiments (see main text).

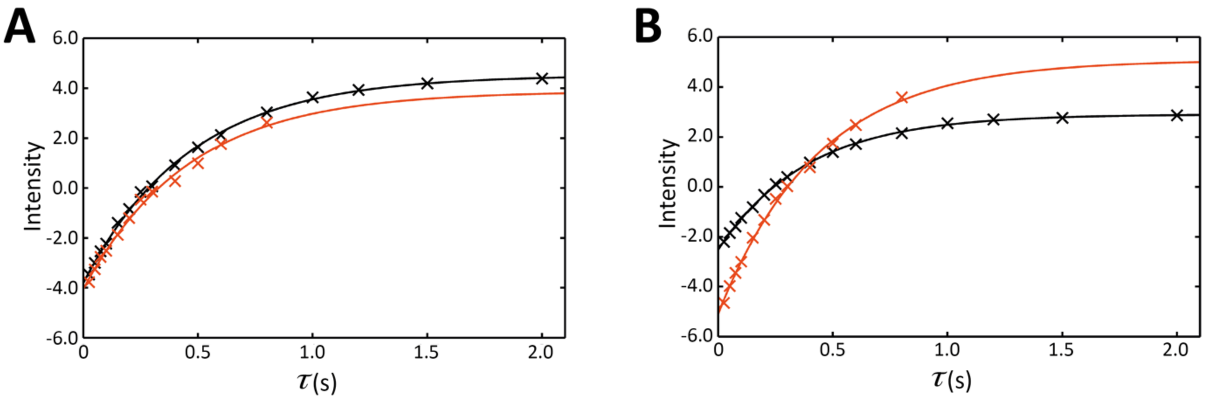


**Figure S4. Longitudinal relaxation of PimA$^{R144C\text{-}TFA}$.** *A*. Intensity in dependence of the relaxation delay τ for the active state resonance in the absence (black) and in the presence of saturating concentrations of GDP-Man (orange). *B*. Intensity in dependence of the relaxation delay τ for the inactive state resonance in the absence (black) and in the presence of saturating concentrations of GDP-Man (orange). Experiments were conducted with 100 μM PimA$^{R144C\text{-}TFA}$ and 500 μM GDP-Man where applicable.

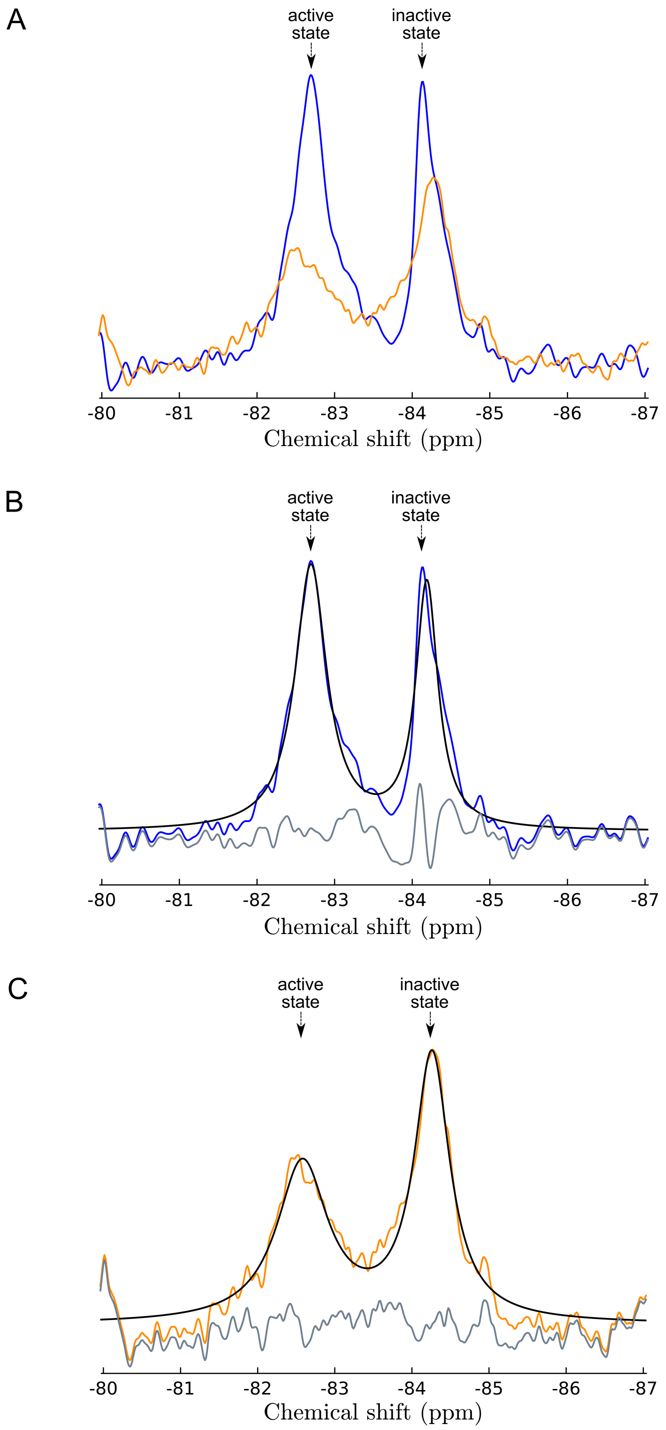


**Figure S5.** *A*. $^{19}$F spectra of PimA$^{\mathrm{R144C\text{-}TFA}}$ in the absence (blue) and presence of saturating concentrations of GDP-Man (orange) acquired at 14.1 T ($^{19}$F frequency of 564 MHz). Experiments were conducted with 100 µM PimA$^{\mathrm{R144C\text{-}TFA}}$ and 500 µM GDP-Man where applicable. *B*. Lorentzian deconvolution of the $^{9}$F spectrum of PimA$^{\mathrm{R144C\text{-}TFA}}$ in the absence of GDP-Man (black). The difference between spectrum and fit in is shown in grey. *C*. Lorentzian deconvolution of the $^{9}$F spectrum of PimA$^{\mathrm{R144C\text{-}TFA}}$ in the presence of GDP-Man (black). The difference between spectrum and fit in is shown in grey.

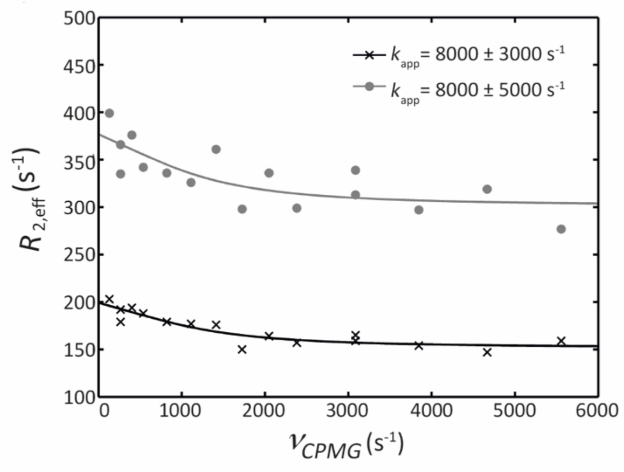


**Figure S6**. Relaxation dispersion for the inactive state resonance of $\mathrm{PimA}^{R144C\text{-}TFA}$ (black) and of $\mathrm{PimA}^{T126C\text{-}V359C\text{-}R144C\text{-}TFA}$ (grey).

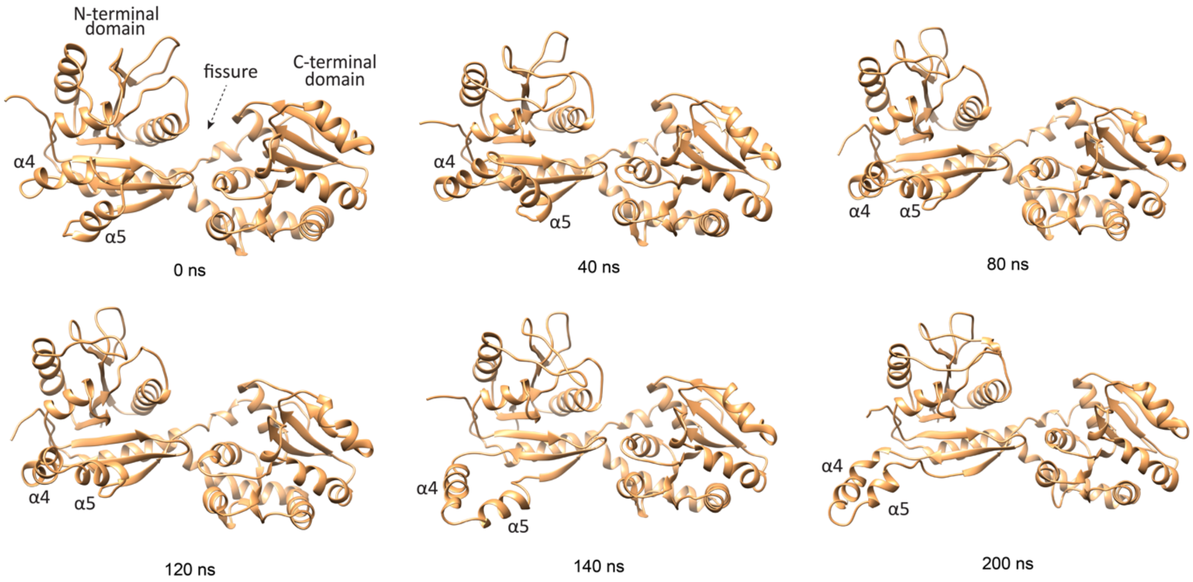


**Figure S7. Conformational shift from compact-inactive to extended-inactive PimA by MD simulations.** Selected frames of PimA extracted from 200 ns steered MD simulations using as starting structure the compact-inactive state.

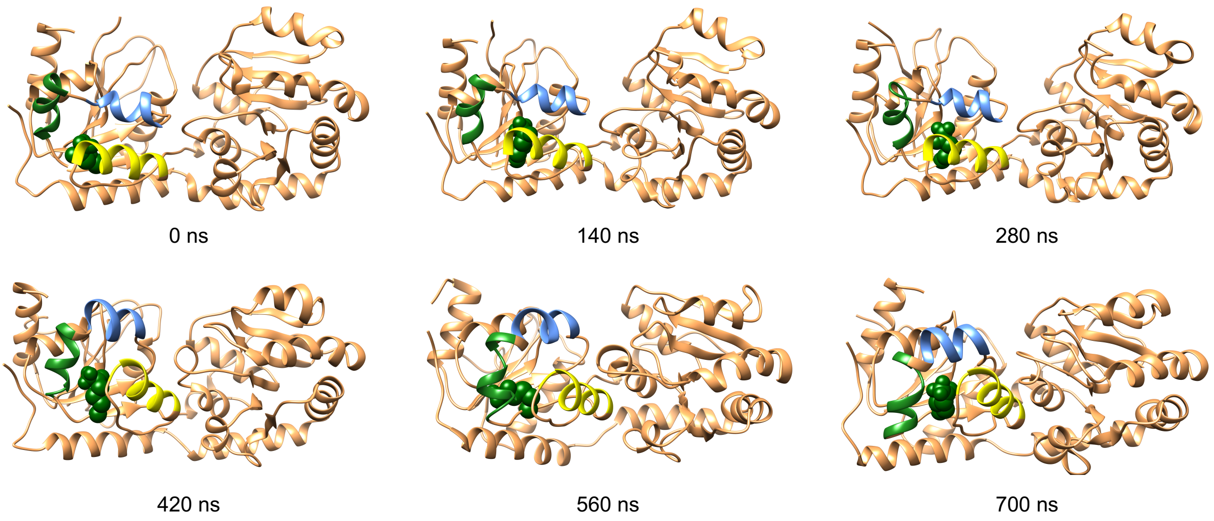


**Figure S8. Conformational shift from compact to a 'more extended' active state of PimA labeled with TFA by MD simulations.** Selected frames of PimA labeled with TFA extracted from 0.7 μs accelerated MD simulations using as starting structure the compact, active state. GDP-Man was not considered during the simulations. The TFA label is shown in green spheres. The helices $\alpha 4_1$, $\alpha 4_2$ and $\alpha 5$ observed in the compact active state (pdb codes 2GEJ and 2GEK; 24) are shown in blue, green and yellow, respectively. Hydrogen atoms have been removed for clarity.

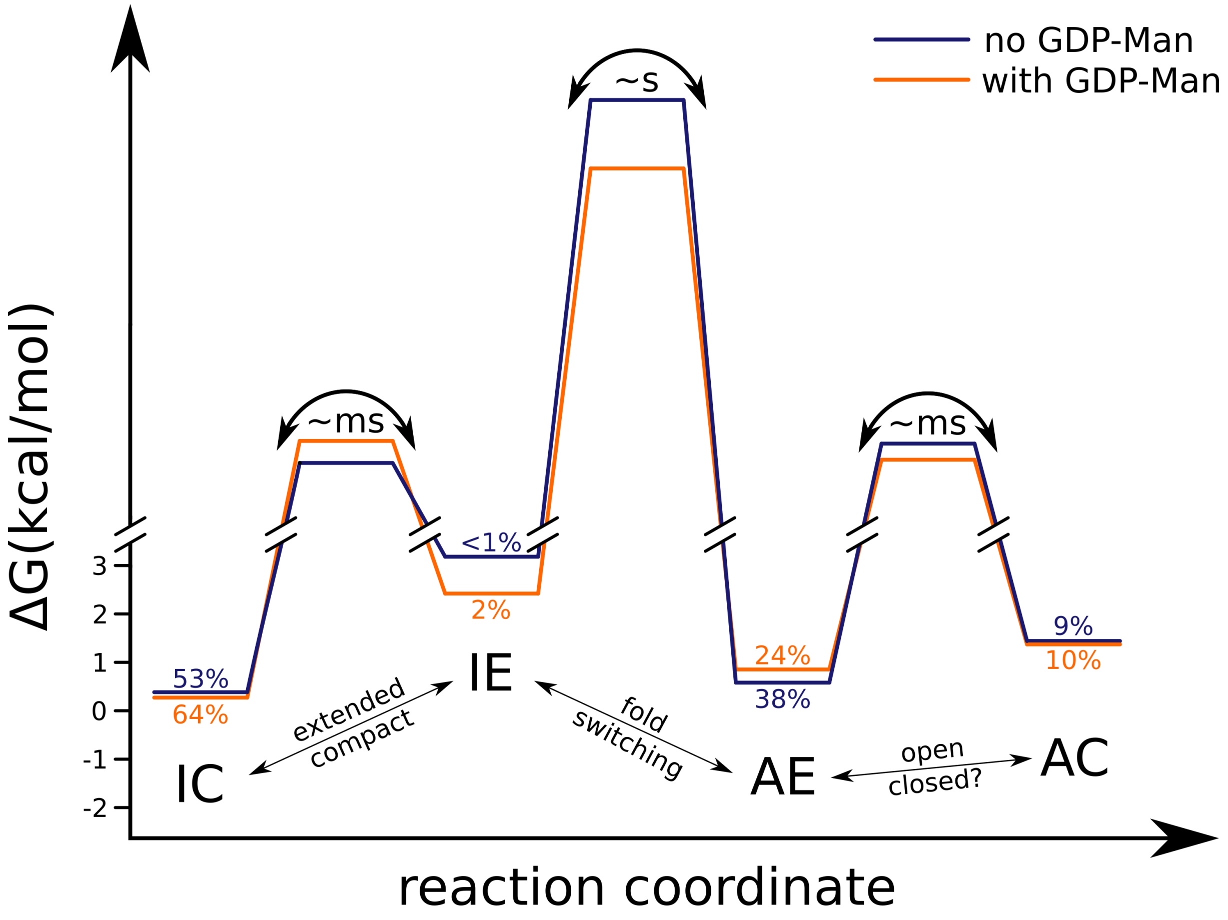


**Figure S9. Free energy diagram of the four states of PimA in the absence of GDP-Man (blue) and in the presence of saturating amounts of GDP-Man (orange)**. States are labeled as: IC – inactive, compact; IE – inactive extended; AE – active, extended; and AC – active compact. The populations are indicated for each state and the order of magnitude of the exchange rates are indicated for the transition states. Populations and free energy differences are derived from data in Table 1 and 2.

# SUPPLEMENTARY TABLES

**Table S1.** Unfolding parameters of wild-type PimA, $PimA^{R144C}$ and $PimA^{R144C\text{-}TFA}$ bound to GDP, measured by CD.

| | $T_m$ (ºC) | ΔH (kcal/mol) |
|---|---|---|
| **$PimA^{WT}$** | 36.8 | 74.5 |
| **$PimA^{WT\text{-}GDP}$** | 48.9 | 222 |
| **$PimA^{R144C}$** | 36.4 | 49 |
| **$PimA^{R144C\text{-}GDP}$** | 46.9 | 190 |
| **$PimA^{R144C\text{-}TFA}$** | 36.4 | 48 |
| **$PimA^{R144C\text{-}TFA\text{-}GDP}$** | 46.3 | 208 |

**Table S2.** Longitudinal relaxation rates $R_1$ ($=T_1^{-1}$) obtained for PimA$^{R144C\text{-}TFA}$ resonances in the absence and presence of saturating concentrations of GDP-Man. Experiments were conducted with 100 μM PimA$^{R144C\text{-}TFA}$ and 500 μM GDP-Man where applicable.

| Condition | Conformer | Chemical shift (ppm) | T1 (ms) | $R_1$ ($s^{-1}$) |
|---|---|---|---|---|
| **Unliganded** | active | -82.8 | 460±10 | 2.16±0.06 |
| **GDP-Man** | active | -82.6 | 460±60 | 2.2±0.3 |
| **Unliganded** | inactive | -84.1 | 390±6 | 2.56±0.04 |
| **GDP-Man** | inactive | -84.3 | 440±30 | 2.3±0.2 |

**Table S3.** Populations of the active and inactive state of $PimA^{R144C\text{-}TFA}$ as obtained from saturation transfer experiments (see Fig. 1 and Table 1 in the main text) and from Lorentzian deconvolutions (see Fig. S5).

| Condition | Conformer | Populations | |
|---|---|---|---|
| | | Saturation transfer | deconvolution |
| **Unliganded** | active | 47% | 61% |
| **GDP-Man** | active | 34% | 46% |
| **Unliganded** | inactive | 53% | 39% |
| **GDP-Man** | inactive | 66% | 54% |
